# Cytomegalovirus Infection and Treatment in Allogeneic Hematopoietic Stem Cell Transplantation: A Retrospective Study from a Single Institution in an Endemic Area

**DOI:** 10.4274/tjh.2016.0225

**Published:** 2017-06-01

**Authors:** Hsin-Chen Lin, Shao-Min Han, Wen-Li Hwang, Cheng-Wei Chou, Kuang-Hsi Chang, Zhi-Yuan Shi, Chieh-Lin Jerry Teng

**Affiliations:** 1 Taichung Veterans General Hospital, Department of Medicine, Division of Hematology-Medical Oncology, Taichung, Taiwan; 2 China Medical University, Department of Public Health, Taichung, Taiwan; 3 Taichung Veterans General Hospital Division of Infectious Diseases, Department of Medicine, Taichung, Taiwan; 4 Tunghai University, Department of Life Science, Tunghai, Taichung, Taiwan; 5 Chung Shan Medical University Faculty of Medicine, Division of Hematology, Taichung, Taiwan

**Keywords:** Allogeneic hematopoietic stem cell transplantation, Cytomegalovirus, graft-versus-host disease, Taiwan

## Abstract

**Objective:**

Although *Cytomegalovirus* (CMV) infection is a major complication after allogeneic hematopoietic stem cell transplantation (allo-HSCT), the risk factors for CMV reactivation and treatment failure in CMV endemic areas have remained unclear. This study investigated the risk factors for CMV reactivation among allo-HSCT recipients in an area where CMV is highly endemic.

**Materials and Methods:**

Medical records of 82 allo-HSCT recipients from a CMV endemic area were retrospectively reviewed. The patients were stratified into two groups: those with CMV reactivation (n=32) and those without CMV reactivation (n=50). We investigated possible variables associated with CMV reactivation and treatment failure.

**Results:**

Univariate analyses showed that non-remission disease status [hazard ratio (HR): 2.15; p=0.032] and ≥grade III acute graft-versus-host disease (GVHD) (HR: 3.07; p=0.002) were associated with CMV reactivation. Multivariate analysis further demonstrated that older age (HR: 1.03; p=0.029) and ≥grade III acute GVHD (HR: 2.98; p=0.012) were associated with CMV reactivation. Overall survival time seemed lower among patients with CMV reactivation than among patients without CMV reactivation, although the difference was not statistically significant (p=0.165). The absence of ≥grade III acute GVHD was associated with successful CMV treatment in the current study (odds ratio: 4.40; p=0.008).

**Conclusion:**

Prophylactic anti-CMV therapy might need to be considered for allo-HSCT recipients who have ≥grade III GVHD.

## Abstract (tr)

**Amaç:**

Sitomegalovirüs (CMV) enfeksiyonu, allojeneik kök hücre transplantasyonu (allo-KHT) sonrası majör bir komplikasyon olmasına rağmen CMV’nin endemik olduğu alanlarda CMV reaktivasyonu ve tedavi başarısızlığı için risk faktörleri belirsizliğini korumaktadır. Bu çalışma CMV’nin büyük ölçüde endemik olduğu bir alanda allo-KHT alıcılarında CMV reaktivasyonu için risk faktörlerini araştırmıştır.

**Gereç ve Yöntemler:**

CMV endemik olduğu bir alandan 82 allo-KHN alıcısının tıbbi kayıtları retrospektif olarak incelendi. Hastalar iki gruba ayrıldı: CMV reaktivasyonu olan (n=32) ve CMV reaktivasyonu olmayan (n=50). CMV reaktivasyonu ve tedavi başarısızlığı ile ilişkili olası değişkenler araştırıldı.

**Bulgular:**

Tek değişkenli analiz CMV reaktivasyonunun remisyon-olmayan hastalık durumu [risk oranı (RO): 2,15; p=0,032) ve ≥grade III akut graft versus host hastalığı (GVHH) (RO: 3,07; p=0,002) ile ilişkili olduğunu gösterdi. Çok değişkenli analiz ayrıca CMV reaktivasyonunun ileri yaş (RO: 1,03; p=0,029) ve ≥grade III akut GVHH (RO: 2,98; p=0,012) ile ilişkili olduğunu gösterdi. Genel sağkalım CMV reaktivasyonu olan hastalarda CMV reaktivasyonu olmayan hastalardan daha düşük görünmekle birlikte fark istatistiksel olarak anlamlı değildi (p=0,165). Bu çalışmada, ≥grade III akut GVHH yokluğu başarılı CMV tedavisi ile ilişkili idi (olasılık oranı: 4,40; p=0,008).

**Sonuç:**

Profilaktik anti-CMV tedavisinin ≥grade III GVHH olan allo-KHT alıcılarında dikkate alınması gerekebilir.

## INTRODUCTION

Allogeneic hematopoietic stem cell transplantation (allo-HSCT) not only improves survival times in patients with acute myeloid leukemia [1] and acute lymphoid leukemia [2], but may also be the only curative therapy for very severe aplastic anemia [3]. Nonetheless, the morbidity and mortality that are associated with allo-HSCT limit its clinical application and efficacy. In addition to graft-versus-host disease (GVHD), infection remains one of the most important complications after allo-HSCT [4]. The incidence of each infection in allo-HSCT recipients varies depending on the time since transplantation. During the neutropenic phase, the principal pathogens are bacteria and *Candida* species. In contrast, *Cytomegalovirus* (CMV) reactivation is the major infectious complication between 30 and 100 days after transplantation. Infections in the late phase are relatively heterogeneous, which is associated with the presence and severity of GVHD [5].

Among the different infectious complications that occur in patients undergoing allo-HSCT, the clinical entity of CMV infection is unique. Reactivation of CMV appears in 60% of seropositive allo-HSCT recipients. Without appropriate treatment, asymptomatic CMV reactivation eventually progresses to symptomatic CMV diseases, which can result in death, especially in immunocompromised hosts. Typically, CMV mainly affects the lungs and gastrointestinal tract [6]. However, CMV retinitis is also common, occurring in 5% of high-risk pediatric allo-HSCT recipients [7].

Although the incidence of symptomatic CMV diseases has decreased significantly because of universal prophylaxis or preemptive therapy, this life-threatening complication still develops in 30% of all allo-HSCT recipients [8]. In addition, CMV seroprevalence is quite endemic [9]. The strategies of CMV prophylaxis and treatment can be entirely different for allo-HSCT recipients in CMV endemic areas and those in non-endemic areas. Moreover, it remains unclear whether ganciclovir in combination with CMV immunoglobulin is more effective than ganciclovir alone for the treatment of CMV reactivation; further investigation is necessary.

We conducted this retrospective study to address these issues, specifically by investigating the risk factors for CMV reactivation among allo-HSCT recipients in an area where CMV is highly endemic. We additionally compared the overall survival (OS) time in patients with and without CMV reactivation. Finally, factors associated with CMV treatment failure were also analyzed.

## MATERIALS AND METHODS

### Patients

The review board of Taichung Veterans General Hospital approved this study. According to the regulations of the institutional review board, informed consent was not required from the patients because of the retrospective study design. Medical records were evaluated for 86 consecutive ≥18-year-old patients who received allo-HSCT at our institution for various hematological diseases from February 2010 to November 2015. Patients without regular follow-up (n=2) and those who died before successful engraftment (n=2) were excluded. The remaining 82 patients were included in the analyses of this study. The median follow-up time for these 82 patients was 513 days (range: 23 to 2045 days). The clinical characteristics of all of the patients are shown in Table 1. The mean ± standard deviation age of our study cohort was 41.98±14.57 years. Acute myeloid leukemia (47.6%) was the major underlying disease that required allo-HSCT. Regarding CMV serostatus, 92.68% (76/82) of recipients were CMV-seropositive before allo-HSCT, while 85.37% (70/82) of donors were CMV-seropositive. Complete remission could not be defined in patients with aplastic anemia (n=9) and chronic myeloid leukemia (n=3). The median OS time was not reached in this study cohort.

### Conditioning Regimen

In this study, the non-myeloablative conditioning regimen was provided irrespective of the patient’s underlying disease. It consisted of total body irradiation (TBI) (200 cGy, day -7) and the administration of fludarabine (30 mg/m^2^/day, from day -6 to day -2) and cyclophosphamide (10 mg/kg/day, from day -5 to day -2). As compared with the non-myeloablative regimen, the myeloablative regimens in the current study were relatively heterogeneous. A TBI-based conditioning regimen (TBI: 1200 cGy, 6 fractions, from day -6 to day -4; cyclophosphamide: 60 mg/kg/day, from day -3 to day -2) was used for patients with acute lymphoblastic leukemia. BuCy_2_ was routinely delivered to patients with acute myeloid leukemia, myelodysplasia syndrome, or chronic myeloid leukemia [10]. Lymphoma patients who received a myeloablative preparative regimen were conditioned using BEAM [11]. In terms of haploidentical transplantation, we followed the Johns Hopkins protocol [12].

### Graft-Versus-Host Disease Prophylaxis

We used cyclosporine as the major immunosuppressant. A trough level of 150-250 ng/mL was the targeted concentration. Myfortic acid was used since day -2 at a dose of 720 mg twice daily and was generally discontinued on day 60. With the exception of the patients undergoing haploidentical transplantation, patients received short-course methotrexate at 15 mg/m^2^ on day 1 and 10 mg/m^2^ on days 3, 6, and 11. Antithymoglobulin (ATG) was routinely given to patients without matched sibling donors at 2 mg/kg/day from day -4 to -2.

### *Cytomegalovirus* Monitoring and Treatment

Our allo-HSCT protocol did not contain CMV antiviral prophylaxis. Because Taiwan is an endemic area for CMV infection, blood donation volunteers do not routinely check their CMV serostatus. Since getting CMV-negative blood products is difficult, to avoid further CMV infection, all allo-HSCT recipients in our study only received leukodepleted and irradiated blood products if blood transfusion was needed. We used quantitative polymerase chain reaction with a COBAS AmpliPrep/COBAS TaqMan CMV system (Roche Molecular Systems, Inc., Branchburg, NJ, USA) to detect the serum CMV viral load. The cutoff of negativity was set as <3333 copies/mL after internal adjustment and validation. Serum CMV viral load was generally monitored once every 1 to 2 weeks. CMV treatment with ganciclovir at 5 mg/kg/day was initiated preemptively in patients with a serum CMV viral load of >3333 copies/mL or with symptomatic CMV infection. Further, 81.3% (26/32) of patients with CMV reactivation also received CMV immunoglobulin simultaneously at a dose of 10,000 units per day on every other day, for a total of five doses. CMV treatment was defined as having been successful if the symptoms of CMV infection disappeared completely and the serum viral load became negative.

### Efficacy Assessments

The patients were stratified into two groups: those with CMV reactivation (n=32) and those without CMV reactivation (n=50). Clinical characteristics, causes of death, and OS were compared between these two groups of patients. We attempted to identify variables associated with CMV reactivation and the failure of CMV treatment. Only the first episode of CMV reactivation was investigated in the current study.

### Statistical Analysis

Student t-tests and Fisher exact tests were used to compare nominal and ordinal variables between patients with and without CMV reactivation. Differences in OS were assessed using the log-rank test. Cox proportional hazards regression was used to investigate variables that were potentially associated with CMV reactivation and successful CMV treatment, as quantified in terms of hazard ratios (HRs) and accompanying 95% confidence intervals (CIs). Data are summarized as mean ± standard deviation where appropriate. P<0.05 was regarded as indicating statistical significance. All statistical analyses were conducted using SPSS 20.0 (IBM Corp., Armonk, NY, USA).

## RESULTS

### Comparisons of Clinical Characteristics between Patients with and without *Cytomegalovirus* Reactivation

Patients without CMV reactivation were younger than patients with CMV reactivation (38.74±13.29 years vs. 47.03±15.23 years; p=0.011). Additionally, more patients in the no-CMV-reactivation group received myeloablative conditioning regimens (78.0% vs. 53.1%; p=0.032). Sex (p=0.899), underlying diseases (p=0.951), donor types (p=0.332), CMV serology status (p=0.176), ATG use (p=0.076), presence of ≥grade III acute GVHD (p=0.940), and disease status before allo-HSCT (p=0.101) did not differ significantly between the two groups of patients (Table 1).

### Risk Factors Associated with *Cytomegalovirus* Reactivation

The average time until the first CMV reactivation was 42.1±31.0 days. Univariate analyses showed that a disease status of non-complete remission (HR: 2.15; 95% CI: 1.07 to 4.43; p=0.032) and ≥grade III acute GVHD (HR: 3.07; 95% CI: 1.53 to 6.16; p=0.002) were associated with CMV reactivation. Multivariate analysis further demonstrated that older age (HR: 1.03; 95% CI: 1.00 to 1.06; p=0.029) and ≥grade III acute GVHD (HR: 2.98; 95% CI: 1.27 to 6.95; p=0.012) were significantly associated with increased risks of CMV reactivation (Table 2).

### Patients with *Cytomegalovirus* Reactivation Had a Trend Toward Inferior Overall Survival

**Figure 1:**
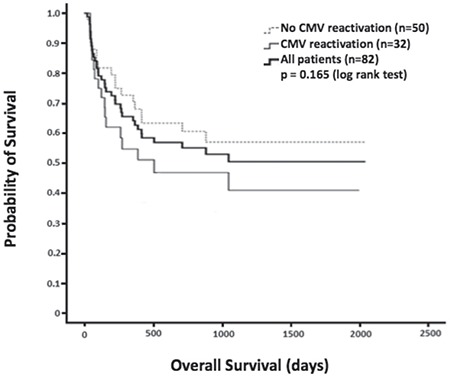
The median overall survival time for patients with Cytomegalovirus (CMV) reactivation was 508 days. However, the median survival time was not reached during the follow-up period for patients without CMV reactivation. Although a trend toward inferior overall survival was observed in patients with CMV reactivation, the difference was not statistically significant (p=0.165).

The median OS time for patients with CMV reactivation was 508 days. However, median OS time was not reached during the follow-up period in patients without CMV reactivation. Although a trend toward inferior OS times was observed in patients with CMV, the difference was not significantly significant (p=0.165) (Figure 1).

### Cause of Death Analyses

Overall, 36 patients (43.9%) died in our study cohort. The mortality rates of patients with and without CMV reactivation were 53.1% (17/32) and 38.0% (19/50), respectively. For patients with CMV reactivation, CMV infection remained the major cause of death, accounting for 41.2% (7/17) of deaths. In contrast, 52.6% of deaths in patients without CMV reactivation were attributable to relapse of the underlying disease. The average OS time was 142.4±92.5 days among patients who died of CMV infection and 285.6±210.7 days among patients who died of their underlying diseases (Table 3).

### Variables Associated with Successful *Cytomegalovirus* Infection Treatment

Because of the limited number of patients in the study cohort, we only conducted univariate analyses, the results of which are shown in Table 4. Briefly, the absence of ≥grade III acute GVHD was the only variable that was significantly associated with successful CMV treatment in the current study (odds ratio: 4.40; 95% CI: 1.48 to 13.15; p=0.008). Notably, use of CMV immunoglobulin was not significantly associated with CMV treatment success (odds ratio: 2.57; 95% CI: 0.58 to 11.50; p=0.217).

## DISCUSSION

CMV serostatus is the most important factor for CMV reactivation in patients undergoing allo-HSCT [13]. In an analysis of a Portuguese cohort that comprised 85.81% CMV-seropositive recipients and 73.27% CMV-seropositive donors, Sousa et al. [14] observed that 60.3% of patients developed CMV infection after allo-HSCT. In our study, 92.68% of recipients and 85.37% of donors were CMV-seropositive, but the incidence of CMV reactivation was only 39.02%. One possible explanation for this lower incidence of CMV reactivation could be the lower incidence of acute GVHD in the present study. Acute GVHD occurred in 70.8% of patients in the Portuguese cohort, yet only 20.73% (17/82) of our patients had ≥grade III acute GVHD. Moreover, among all of the variables investigated in the present study, ≥grade III acute GVHD was the only variable that was a significant risk factor for CMV reactivation in both univariate (p=0.002) and multivariate (p=0.012) analyses (Table 2). Our result is confirmed by a study of Cohen et al. [15], in which GVHD was also identified as a risk factor for first CMV reactivation in allo-HSCT recipients (HR=5.091, p=0.021).

In addition to GVHD, conditioning regimens could be also associated with CMV reactivation in patients receiving allo-HSCT. However, evidence concerning the association between conditioning regimens and CMV reactivation has been inconsistent. Although Cohen et al. [15] identified the myeloablative preparative regimen as a risk factor, George et al. [13] considered the non-myeloablative regimen to be an independent predictor of CMV reactivation. Interestingly, the type of preparative regimen did not correlate with CMV reactivation in our study. Additionally, our data did not demonstrate an association between the use of ATG and CMV reactivation, either. This result, however, is not consistent with the prior findings of Wu et al. [16]. Small study cohort and short follow-up time could be among the reasons for these inconsistent findings, suggesting that further studies are required.

Previous studies also investigated whether CMV reactivation results in inferior outcomes in allo-HSCT patients. Sousa et al. [14] found that CMV infection was associated with inferior median post-transplantation survival in allo-HSCT recipients. However, the data from our cohort did not show a statistically significant difference in OS between patients with and without CMV reactivation. This result is similar to the prior findings of Liu et al. [17], who found no survival disadvantage for patients with CMV infection (p=0.699) or CMV disease (p=0.093). Notably, CMV infection is not entirely a poor prognostic predictor for allo-HSCT. On the contrary, CMV reactivation is considered to reduce the risk of relapse in patients with acute myeloid leukemia after allo-HSCT [18,19]. This protective effect might partially reverse the inferior outcomes of CMV reactivation in patients undergoing allo-HSCT. Additionally, the good efficacy of CMV treatment could potentially also be responsible for the non-inferiority of OS in patients with CMV infection. In our study cohort, the rate of successful CMV treatment for the first reactivation was as high as 87.50%.

In terms of CMV treatment, although the rates of clearance of CMV viremia are similar with oral valganciclovir and ganciclovir in the post-allo-HSCT population [20], ganciclovir remains the drug of choice in our allo-HSCT setting. Moreover, most of the patients with CMV reactivation (81.3%) in our study cohort were treated with CMV immunoglobulin simultaneously. However, whether CMV immunoglobulin could facilitate successful CMV treatment remains controversial. A study by Ranganathan et al. [21] revealed that prophylactic CMV immunoglobulin decreased risk of CMV infection, but not CMV disease, after lung transplantation in pediatric patients. The univariate analyses of our data also did not demonstrate a significant association between the use of CMV immunoglobulin and the success of CMV treatment (odds ratio: 2.57; 95% CI: 0.58 to 11.50; p=0.217). More studies are required to identify the role and efficacy of CMV immunoglobulin in allo-HSCT.

Among the 32 patients with first CMV reactivation in our study cohort, treatment failure occurred in four patients (4/32, 12.50%). This result raises another important issue: the identification of individuals who are at higher risk of treatment failure and possibly require CMV prophylaxis. CMV prophylaxis by valganciclovir appears to fail as a means of improving CMV disease-free and invasive infection-free survival in allo-HSCT recipients [22]. A phase 2 study by Chemaly et al. [23] showed that letermovir prophylaxis effectively reduced the incidence of CMV infection after allo-HSCT. Its impact on OS, however, was not clear. CMV antiviral prophylaxis was not part of the routine of our allo-HSCT protocol. Notably, our results showed that patients with ≥grade III GVHD were more refractory to CMV treatment (odds ratio: 4.40; 95% CI: 1.48 to 13.15; p=0.008), suggesting that patients with ≥grade III GVHD could potentially benefit from valganciclovir prophylaxis. However, more evidence is needed before jumping to any conclusions.

The major limitations of the current study are its retrospective study design and the small number of patients. In addition, the active immunosuppressants of each patient at the time at which either CMV or GVHD occurs are very heterogeneous. The current study could not precisely identify the impact of immunosuppression on CMV reactivation. Moreover, the role of CMV immunoglobulin in either preemptive or targeted treatment remains uncertain after our analyses. Studies with prospective and randomized-control designs, focusing on more particular clinical scenarios, are urgently needed for this unmet clinical need.

## CONCLUSION

Older age, non-complete remission disease status, and ≥grade III GVHD were risk factors for CMV reactivation in allo-HSCT recipients. The presence of ≥grade III acute GVHD could be associated with CMV treatment failure. Prophylactic anti-CMV therapy needs to be considered in allo-HSCT recipients with ≥grade III GVHD.

## Figures and Tables

**Table 1:**
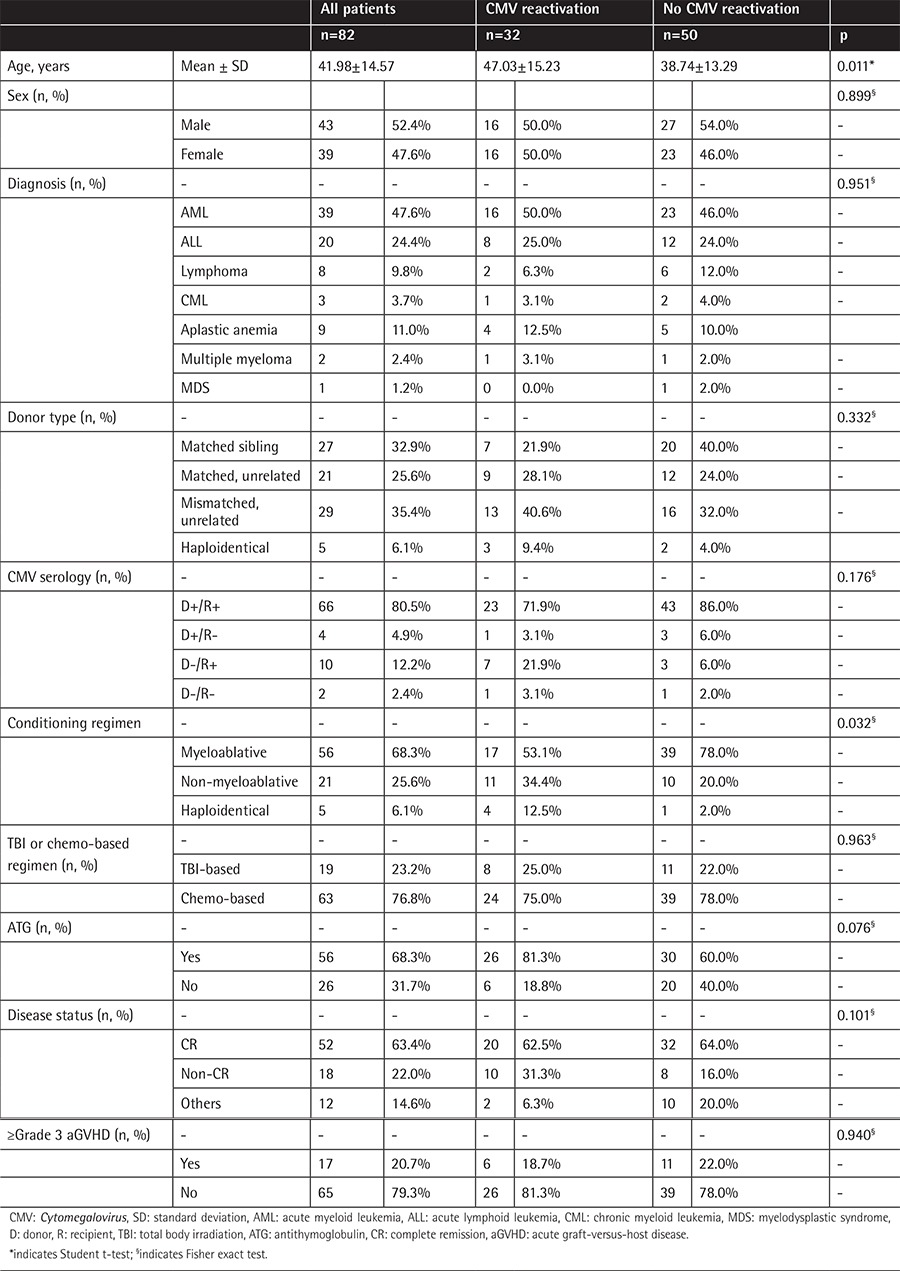
Comparisons of clinical characteristics between patients with and without *Cytomegalovirus* reactivation.

**Table 2:**
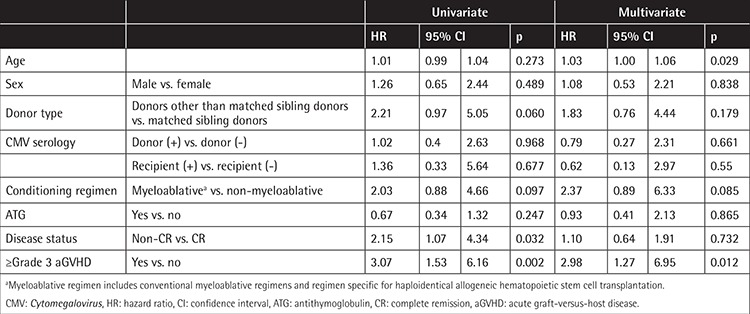
Risk factors associated with *Cytomegalovirus* reactivation.

**Table 3:**
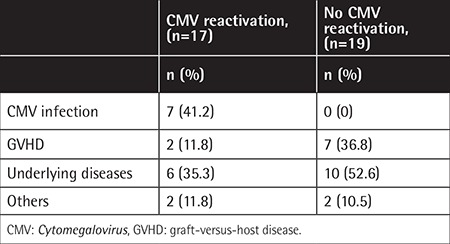
Causes of death in patients undergoing allogeneic hematopoietic stem cell transplantation.

**Table 4:**
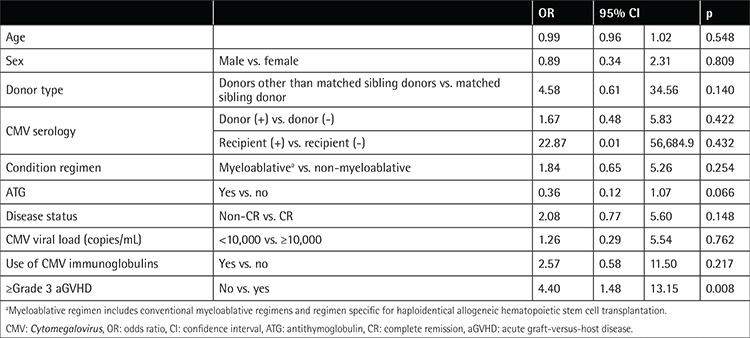
Variables associated with successful *Cytomegalovirus* treatment by univariate analysis (n=32).

## References

[1] Ho AD, Schetelig J, Bochtler T, Schaich M, Schafer-Eckart K, Hanel M, Rösler W, Einsele H, Kaufmann M, Serve H, Berdel WE, Stelljes M, Mayer J, Reichle A, Baldus CD, Schmitz N, Kramer M, Rollig C, Bornhauser M, Thiede C, Ehninger G, Study Alliance Leukemia (2016). Allogeneic stem cell transplantation improves survival in patients with AML characterized by a high allelic ratio of mutant FLT3-ITD. Biol Blood Marrow Transplant.

[2] Chalandon Y, Thomas X, Hayette S, Cayuela JM, Abbal C, Huguet F, Raffoux E, Leguay T, Rousselot P, Lepretre S, Escoffre-Barbe M, Maury S, Berthon C, Tavernier E, Lambert JF, Lafage-Pochitaloff M, Lheritier V, Chevret S, Ifrah N, Dombret H, Group for Research on Adult Acute Lymphoblastic Leukemia (GRAALL) (2015). Randomized study of reduced-intensity chemotherapy combined with imatinib in adults with Ph-positive acute lymphoblastic leukemia. Blood.

[3] Cutler C (2014). Timing of allogeneic stem cell transplantation for myelodysplastic syndromes and aplastic anemia. Hematology Am Soc Hematol Educ Program.

[4] Styczynski J, Czyzewski K, Wysocki M, Gryniewicz-Kwiatkowska O, Kolodziejczyk-Gietka A, Salamonowicz M, Hutnik L, Zajac-Spychala O, Zaucha-Prazmo A, Chelmecka-Wiktorczyk L, Siewiera K, Fraczkiewicz J, Malas Z, Tomaszewska R, Irga-Jaworska N, Plonowski M, Ociepa T, Pierlejewski F, Gamrot Z, Urbanek-Dadela A, Gozdzik J, Stolpa W, Dembowska-Baginska B, Perek D, Matysiak M, Wachowiak J, Kowalczyk J, Balwierz W, Kalwak K, Chybicka A, Badowska W, Szczepanski T, Drozynska E, Krawczuk-Rybak M, Urasinski T, Mlynarski W, Woszczyk M, Karolczyk G, Sobol-Milejska G, Gil L, Polish Society of Paediatric Oncology and Haematology (2016). Increased risk of infections and infection-related mortality in children undergoing haematopoietic stem cell transplantation in comparison to conventional anticancer therapy: a multicentre nationwide study. Clin Microbiol Infect.

[5] Gratwohl A, Brand R, Frassoni F, Rocha V, Niederwieser D, Reusser P, Einsele H, Cordonnier C, Acute and Chronic Leukemia Working Parties; Infectious Diseases Working Party of the European Group for Blood and Marrow Transplantation (2005). Cause of death after allogeneic haematopoietic stem cell transplantation (HSCT) in early leukaemias: an EBMT analysis of lethal infectious complications and changes over calendar time. Bone Marrow Transplant.

[6] Garrido RS, Aguado JM, Diaz-Pedroche C, Len O, Montejo M, Moreno A, Gurgui M, Torre-Cisneros J, Pareja F, Segovia J, Garcia M, Lumbreras C (2006). A review of critical periods for opportunistic infection in the new transplantation era. Transplantation.

[7] Hiwarkar P, Gajdosova E, Qasim W, Worth A, Breuer J, Chiesa R, Ridout D, Edelsten C, Moore A, Amrolia P, Veys P, Rao K (2014). Frequent occurrence of cytomegalovirus retinitis during immune reconstitution warrants regular ophthalmic screening in high-risk pediatric allogeneic hematopoietic stem cell transplant recipients. Clin Infect Dis.

[8] Pollack M, Heugel J, Xie H, Leisenring W, Storek J, Young JA, Kukreja M, Gress R, Tomblyn M, Boeckh M (2011). An international comparison of current strategies to prevent herpesvirus and fungal infections in hematopoietic cell transplant recipients. Biol Blood Marrow Transplant.

[9] Cannon MJ, Schmid DS, Hyde TB (2010). Review of cytomegalovirus seroprevalence and demographic characteristics associated with infection. Rev Med Virol.

[10] Andersson BS, Kashyap A, Gian V, Wingard JR, Fernandez H, Cagnoni PJ, Jones RB, Tarantolo S, Hu WW, Blume KG, Forman SJ, Champlin RE (2002). Conditioning therapy with intravenous busulfan and cyclophosphamide (IV BuCy2) for hematologic malignancies prior to allogeneic stem cell transplantation: a phase II study. Biol Blood Marrow Transplant.

[11] Przepiorka D, Besien K, Khouri I, Hagemeister F, Samuels B, Folloder J, Ueno NT, Molldrem J, Mehra R, Körbling M, Giralt S, Gajewski J, Donato M, Cleary K, Claxton D, Braunschweig I, Andersson B, Anderlini P, Champlin R (1999). Carmustine, etoposide, cytarabine and melphalan as a preparative regimen for allogeneic transplantation for high-risk malignant lymphoma. Ann Oncol.

[12] Bolanos-Meade J, Fuchs EJ, Luznik L, Lanzkron SM, Gamper CJ, Jones RJ, Brodsky RA (2012). HLA-haploidentical bone marrow transplantation with posttransplant cyclophosphamide expands the donor pool for patients with sickle cell disease. Blood.

[13] George B, Pati N, Gilroy N, Ratnamohan M, Huang G, Kerridge I, Hertzberg M, Gottlieb D, Bradstock K (2010). Pre-transplant cytomegalovirus (CMV) serostatus remains the most important determinant of CMV reactivation after allogeneic hematopoietic stem cell transplantation in the era of surveillance and preemptive therapy. Transpl Infect Dis.

[14] Sousa H, Boutolleau D, Ribeiro J, Teixeira AL, Pinho Vaz C, Campilho F, Branca R, Campos A, Baldaque I, Medeiros R (2014). Cytomegalovirus infection in patients who underwent allogeneic hematopoietic stem cell transplantation in Portugal: a five-year retrospective review. Biol Blood Marrow Transplant.

[15] Cohen L, Yeshurun M, Shpilberg O, Ram R (2015). Risk factors and prognostic scale for cytomegalovirus (CMV) infection in CMV-seropositive patients after allogeneic hematopoietic cell transplantation. Transpl Infect Dis.

[16] Wu JL, Ma HY, Lu CY, Chen JM, Lee PI, Jou ST, Yang YL, Chang HH, Lu MY, Chang LY, Huang LM ([Epub ahead of print]). Risk factors and outcomes of cytomegalovirus viremia in pediatric hematopoietic stem cell transplantation patients. J Microbiol Immunol Infect.

[17] Liu YC, Lu PL, Hsiao HH, Chang CS, Liu TC, Yang WC, Lin SF (2012). Cytomegalovirus infection and disease after allogeneic hematopoietic stem cell transplantation: experience in a center with a high seroprevalence of both CMV and hepatitis B virus. Ann Hematol.

[18] Elmaagacli AH, Steckel NK, Koldehoff M, Hegerfeldt Y, Trenschel R, Ditschkowski M, Christoph S, Gromke T, Kordelas L, Ottinger HD, Ross RS, Horn PA, Schnittger S, Beelen DW (2011). Early human cytomegalovirus replication after transplantation is associated with a decreased relapse risk: evidence for a putative virus-versus-leukemia effect in acute myeloid leukemia patients. Blood.

[19] Takenaka K, Nishida T, Asano-Mori Y, Oshima K, Ohashi K, Mori T, Kanamori H, Miyamura K, Kato C, Kobayashi N, Uchida N, Nakamae H, Ichinohe T, Morishima Y, Suzuki R, Yamaguchi T, Fukuda T (2015). Cytomegalovirus reactivation after allogeneic hematopoietic stem cell transplantation is associated with a reduced risk of relapse in patients with acute myeloid leukemia who survived to day 100 after transplantation: The Japan Society for Hematopoietic Cell Transplantation Transplantation-related Complication Working Group. Biol Blood Marrow Transplant.

[20] Chawla JS, Ghobadi A, Mosley J, Verkruyse L, Trinkaus K, Abboud CN, Cashen AF, Stockerl-Goldstein KE, Uy GL, Westervelt P, DiPersio JF, Vij R (2012). Oral valganciclovir versus ganciclovir as delayed pre-emptive therapy for patients after allogeneic hematopoietic stem cell transplant: a pilot trial (04-0274) and review of the literature. Transpl Infect Dis.

[21] Ranganathan K, Worley S, Michaels MG, Arrigan S, Aurora P, Ballmann M, Boyer D, Conrad C, Eichler I, Elidemir O, Goldfarb S, Mallory GB, Mogayzel PJ, Parakininkas D, Solomon M, Visner G, Sweet SC, Faro A, Danziger-Isakov L (2009). Cytomegalovirus immunoglobulin decreases the risk of cytomegalovirus infection but not disease after pediatric lung transplantation. J Heart Lung Transplant.

[22] Boeckh M, Nichols WG, Chemaly RF, Papanicolaou GA, Wingard JR, Xie H, Syrjala KL, Flowers ME, Stevens-Ayers T, Jerome KR, Leisenring W (2015). Valganciclovir for the prevention of complications of late cytomegalovirus infection after allogeneic hematopoietic cell transplantation: a randomized trial. Ann Intern Med.

[23] Chemaly RF, Ullmann AJ, Stoelben S, Richard MP, Bornhauser M, Groth C, Einsele H, Silverman M, Mullane KM, Brown J, Nowak H, Kölling K, Stobernack HP, Lischka P, Zimmermann H, Rubsamen-Schaeff H, Champlin RE (2014). Letermovir for cytomegalovirus prophylaxis in hematopoietic-cell transplantation. N Engl J Med.

